# Cybersecurity: a critical priority for digital mental health

**DOI:** 10.3389/fdgth.2023.1242264

**Published:** 2023-09-14

**Authors:** Becky Inkster, Catherine Knibbs, Maria Bada

**Affiliations:** ^1^ Department of Psychiatry, Cambridge University, Cambridge, United Kingdom; ^2^ School of Health Sciences, Salford University, Salford, United Kingdom; ^3^ Department of Psychology, Queen Mary University of London, London, United Kingdom

**Keywords:** cybersecurity, mental health, digital mental health, victim support, data protection

## Abstract

There has been a surge in the supply and demand of digital mental health support services in recent times. There have also been high profile cyberattacks specifically targeting mental health and behavioral services, along with a shift toward targeting vulnerable people directly. Cyberattacks involving personal health data, especially sensitive mental health data, could have devastating consequences to vulnerable people, those close to them, and many other stakeholders. This article calls for the immediate examination of the current state of cybersecurity in the digital mental healthcare industry to collectively identify risks and to protect user and provider vulnerabilities. This article points to the need to build a global cybersecurity culture within digital mental health while also working closely with other industries. The article concludes by making some preliminary recommendations to help support the creation of standards that will enhance the collective preparedness for future responses to cybersecurity threats and attacks.

## Introduction

Cybersecurity in digital mental health is being overlooked as a critical priority. For example, in a recent report by the World Economic Forum Governance Toolkit for Digital Mental Health the word “cybersecurity” is mentioned only once in over 70 pages (1). Furthermore, whilst the profession of mental health care is focused on delivery of services, the digital and technical knowledge of those services is not necessarily part of training mental health professionals.

Data breaches involving mental health information can have widespread impacts, for clients, patients, their loved ones, practitioners themselves and even providers of the technology. Therefore, there is an immediate need to examine the current state of cybersecurity in digital mental healthcare to identify risks and to protect user and provider vulnerabilities. This may also include reviewing the membership bodies that oversee healthcare professional practice and training to identify where those risks are present.

This article highlights some of the ways in which many stakeholders can be affected. It also highlights different ways in which providers and other stakeholders can become vulnerable from design and implementation of these services. It then concludes with some preliminary recommendations for helping to cultivate a global cybersecurity culture within the digital mental health space, which would expedite the creation of standards to enhance collective preparedness to respond to future cybersecurity threats and attacks.

Personal health data is now the most valuable form of data on the dark web according to sources (2), and cybersecurity breaches in the healthcare sector continue to grow (3). In January 2021 alone, a total of 878.17 million data records were compromised worldwide, which is more than in all of 2017 (4).

The pandemic has also accelerated the global adoption of digital mental health services (5), but this growth in supply and demand could increase the exposure of sensitive data to cybersecurity threats and attacks. Furthermore, there is a lack of training overall in cybersecurity matters and data protection laws for the professionals using those very services who often, if not always, process what are called “special categories” of data under the Data Protection Act 2018 (6).

Several high-profile cyberattacks have already targeted mental health and behavioral services providers. For example, Finland's largest psychotherapy provider, Vastaamo, declared bankruptcy in 2021 after experiencing data breaches in which confidential therapy session notes, as well as other personal information, were stolen (7). After the company allegedly refused to pay the ransom, the cyber criminals directly targeted victims, including minors (8). This case is an example of how cybercrime has evolved beyond encrypting data to using blackmail and extortion aimed at already vulnerable people with extremely private and sensitive information (9). In another recent high-profile case, SalusCare, the largest provider of behavioral healthcare services in Southwest Florida, USA, filed a lawsuit against Amazon to force the release of allegedly stolen mental health data that was exfiltrated to an Amazon Web Services (AWS) storage account (10, 11). According to SalusCare, the stolen data contains extremely personal and sensitive records of patients’ psychiatric and addiction counselling and treatment, as well as sensitive financial information (10, 11). In a cyberattack on the Behavioral Health Center, Bangor, Maine, USA, highly confidential psychotherapy records were stolen, which included evaluations, session notes, and records of sex offenders and sexual abuse victims (12). This stolen data was listed on the dark web and has allegedly been sold to an unknown party for unknown purposes (12). Cyber-attacks have also targeted private health insurers, NHS mental health trusts, and mental health charities (13–15).

## Impact to victims and those close to them

Data breaches can harm victims in multiple ways, including mental health harms (16, 17). For example, cyber-attacks can potentially trigger or exacerbate issues such as anxiety, insomnia, trauma, paranoia, substance abuse, or even suicidal behaviors and action (18–20) or the repetition of these kinds of “cybertrauma” (21). Attacks can cause interpersonal harms due to disclosures, such as when abusers are named in therapy sessions. They can also cause financial harm that could exacerbate mental health issues and leave victims exposed to fraud and identity theft, or financial hardship if the victim pays a ransom. Reputational harms, especially for high-profile victims, can also occur given that stigma about mental health still exists globally. Loved ones, and those who are close to the victim are also at risk from data breaches, such as when their details are listed as emergency contacts. Surveillance advertising, which may also be a side-effect of tracking is also a harm, whereby data is sold to the market to target those individuals (22) specifically and directly.

Research findings with victims of computer misuse report psychological harm such as anger, anxiety, fear, isolation, and embarrassment (23). In the case of a victim of identity theft after a data breach, victims feel violated, betrayed, vulnerable, angry, and powerless (24). Victims might go into stages of grief and might feel guilty or ashamed. The emotional harm can lead to trauma or physical symptoms such as difficulty in sleeping as mentioned earlier (25). Repeat victims who might have experienced such experiences more than once, might have a unique set of support needs, for both emotional or physical problems such as the need for counselling, and support from the criminal justice system (26).

Targeting minors, who are already at a heightened exploitation risk, could lead to them being manipulated into carrying out illegal hacking activities, becoming “money mules” to perform illegal online financial transactions, or being groomed by online predators to share age-inappropriate and often sexually explicit content; all of which can have implications for young people's mental health and future outcomes (27).

Cyber-attacks not only negatively impact victims of the crime, but they can also impact all other service users who are no longer able to access the support they need from their provider. For example, access to services would be especially important for vulnerable people in crisis and times of need, including high suicidal risk, service disruptions to medication delivery and monitoring medication adherence. Such issues raise questions about how individuals should be supported before, during, and after such events. How do data breaches and service disruptions impact psychological and other clinical outcomes? What actions should a mental health practitioner take when they become aware of such intrusions or disruptions to the system? For example, one study assessing 3,025 hospitals with 14,297 unique hospital-year observations found that after a data breach had occurred clinical outcomes worsened with as many as 36 additional deaths per 10,000 heart attacks (28). In addition, cyber-attacks, such as the WannaCry attack, resulted for many in worry as well as a sense of helplessness (29). When victims have experienced identity theft what happens to their socioeconomic status? A data breach can also weaken or even destroy trust between service users and providers, and ill-prepared cybersecurity breach responses could add to the stigma around seeking and receiving mental health treatments. For example, users might start to avoid or delay seeking treatment, or omit or give false information during assessments thus harming the quality of care.

## Digital mental health providers

Digital mental health providers offer critical and timely support to millions of people around the world, including extremely sensitive support such as suicide safety and prevention planning. This can make digital mental health providers “attractive” targets for cybercriminals. When security-by-design and default is not a priority from the onset, providers risk hurting the very people they are trying to help. Digital support can scale readily, but so too can disruption and devastation. There could be many immediate and long-lasting consequences to mental health services providers after being targeted by cyber threats and attacks. Employees and other stakeholders connected with the service could have their data stolen or sold to third parties or criminals. This can also be a stressful and highly emotional period for all staff, such as provoking panic, anger, and guilt (30) especially for employees who continue to work with the mental health services provider while the breach is being investigated, and especially if the breach involves an employee misstep (clicking on a phishing email) or their own data being breached. From a business perspective, data breaches could lead to serious impacts on revenue including slow operational recovery after a breach as well as irreparable reputational damage. Cybercriminals are increasingly targeting small and medium-sized enterprises (SMEs) and small and medium-sized businesses (SMBs) (31). Many digital mental health services providers are SMEs and SMBs, therefore this could have a serious impact on the provider's sustainability (32), and in extreme cases lead to bankruptcy (7). Even security training for employees does not guarantee improvements in security awareness at the organizational level or in real-world attack situations (33). Furthermore, while cybersecurity insurance offers a mechanism to mitigate some risks to providers, such as underwriting ransomware payments to cybercriminals, it remains uncertain as to what extent providers will be able to shift cybersecurity risks to insurers in the future. For example, the global insurer, AXA, announced a landmark policy decision in 2021 to stop reimbursements of ransomware payments in France (34). Some governments have started to inform companies that they should not pay ransomware (35). From a legal and regulatory perspective, digital mental health providers must be aware of any obligations and repercussions related to data breaches. For example, the US Department of Treasury's Office of Foreign Assets Control (OFAC) warned that businesses could be in violation of USA law if they pay groups that are on the sanctions list (35). Other potential criminal penalties, such as prosecution, fine or imprisonment (36), could come from improperly disclosing a data breach, for example, by concealing security failings. For example, Article 34 of the European General Data Protection Regulation (GDPR) states that data controllers must communicate a data breach to people impacted without undue delay and report to the Information Commissioner's Office within 72 h of becoming aware of such instances (37).

This issue also highlights that practitioners and users of the systems must either use further technology or employ staff to highlight a breach where possible or be able to understand cybersecurity and technology principles with enough competence to recognize when data has been breached. However, many providers find out that criminal activity has taken place when the system becomes unavailable or “locked” due to malware or virus notifications (38).

## The pandemic is shifting boundaries for mental health support and subsequently expanding and evolving the cyber threat attack surface for cybercriminals

The pandemic has brought a profound shift toward the digital provision of mental health support (5), albeit with some exceptions, such as it being ruled illegal to section someone under the Mental Health Act using remote video consultations in the UK (39, 40). In April 2020, an FDA regulatory change allowed digital mental health providers to bypass certain processes (41). While the FDA still required that products meet the same level of cybersecurity protections, including software verification, validation, and hazard analysis, this action by the FDA has still subsequently increased the gap between “time to market and the time to security” as stated by the World Economic Forum, which inferred that digital mental health services providers could be “introducing new vulnerabilities faster than they can be secured” (42).

The pandemic also increased opportunities for adversaries to find vulnerabilities and infiltrate networks, which subsequently has expanded the attack surface. This was in part due to the shift towards remote working, increased internet connectivity, and dependency on digital infrastructures from possibly unprotected or unknown networks. Moreover, many practitioners were at home during this time using their own Internet Service Provider who may not have the same infrastructure of security protocols as a business that provides a room for the practitioner to use, or the home practitioner may not have had their own security systems in place or know how to if they lacked data protection, cybersecurity, and awareness training (43).

During the pandemic, ransomware attacks increased by 62% globally, and by 158% in North America between 2019 and 2021 (44). This increase in cybercrime can have a serious subsequent impact on the mental health of cybersecurity workers. A survey by OneLogin called IAMokay Mental Health Survey polled 250 technology leaders worldwide and found that over three-quarters believed the pandemic had increased workplace stress, 86% reported an increase in workload, and almost a quarter of respondents said they used alcohol, narcotics, or prescription medication to manage their stress (45). Nearly three-quarters of respondents believed their organization cares about their mental health, and nearly half (49%) said their employer has provided access to mental health services (45).

There are many ways that providers can experience cyber threats and attacks, including brute force and distributed denial-of-service (DDoS), credential stuffing, person-in-the-middle and providers can also be made vulnerable in different ways, such as through interactions with clients and supplier third party software vulnerabilities (46, 47). There are also factors such as human error and social engineering at play, including sophisticated spear phishing attacks that exploit cognitive biases and target disgruntled or vulnerable staff members to help cybercriminals steal information or use disguises to enter premises (46–49).

While cybercrime is often driven by financial incentives, “hacktivist” groups might have additional motivations, such as causing disruptions to clinical operations for social purposes. For example, a hacktivist group in the USA had opposing views about how a hospital was managing a patient's case and subsequently targeted the hospital with a DDoS attack (50). This may also be an issue for hacktivists who may take action where services that include “hot” or sensitive topics such as children's services providing gender-based care, sexual health, or areas of reproductive justice, abortion, and pregnancy, which all relate to and fall within the mental health and healthcare paradigm.

Other tactics used by cybercriminals include exploiting web services and Application Programming Interfaces (APIs). APIs define how apps can communicate with other apps and systems, and this form of communication now accounts for more than 83% of all internet traffic (51). According to a Salt Security report, malicious API traffic is growing faster than non-malicious API calls, whereby API calls grew 51% vs. malicious traffic that grew 211% in 2021 (52). Some have even predicted that API attacks would become the most frequent attack vector for application breaches (53). Many high-profile companies have experienced API-related cybersecurity problems, including Peloton Equifax, Instagram, Meta, Amazon and Paypal. According to a report by Salt Security, 91% of companies had API-related security problems (54). Within the digital mental health space, one might speculate a similar degree of risk exists albeit far less reported. One documented API-related security threat involving the digital mental health app provider, “Feelyou”, was reported by The Daily Dot stating that the provider patched a vulnerability that was—until then—leaving nearly 80,000 email addresses of its users exposed online (55).

As the pandemic accelerated the use of mobile healthcare apps, this increased the exposure of health data through API vulnerabilities used by mobile health applications. For example, in the largest unveiling of vulnerabilities in telemedicine APIs, a recent report called “All That We Let In” by ethical hacker Alissa Knight and Approov found that all 30 mobile health apps investigated were vulnerable to API attacks, which collectively exposed 23 million mobile health users (56, 57). While APIs play a crucial role in supporting health IT interoperability by allowing multiple data sources to become transferable and to help providers give better care (58) much more research is needed, especially as to the security of Fast Healthcare Interoperability Resources (FHIR) standards and compliant applications, and potential FHIR API vulnerabilities (59–61).

## Recommendations to remediate and respond to cyber threats and attacks

All digital mental health providers are vulnerable to cyber threats and attacks. How can we be individually and collectively more responsive and resilient in the face of those attacks so that the consequences are minimized? While improving the security of systems is vital, there are other factors to address, such as creating standards for victim support after breaches, and building an evidence base to evaluate the impact of data breaches on clinical and psychological outcomes. These goals will be best achieved by working collaboratively, and we make the following preliminary recommendations:

1. Training

Cybersecurity and digital mental health training in the curriculums of mental health professional schools must be ensured, especially when mental health practitioners begin their professional studies. Many students begin at the lower college levels, who are often collecting special categories of data, processing and controlling this and in some cases carrying this data with them to and from educational settings, or sending to their respective tutors to be assessed, with little knowledge of the systems in place that protect this data and systems. Training should follow the legislations and law as discussed earlier in this article and become their ethical “privacy by design and default approach to practice”. This will include understanding cybersecurity of their own technology such as smartphones, tablets, and computers that they use to collect all forms of data and access the platforms and spaces of digital metal health. Training should help professionals to become aware of cybersecurity issues that surround services that they use and should provide education on how to protect both themselves and their service users from the data breaches.

2. Develop a global cybersecurity culture in digital mental health

Digital mental health providers, especially those with an international user base, must navigate a growing number and increasingly complex system of regulations and standards, such as the DPA (8), GDPR (62), soon PECR (63), ePR (64), DSA (65), OSB (66), CCPA (67), the Cybersecurity Law of the People's Republic of China (68) and many others worldwide. This makes it harder to keep up with cyber criminals who do not comply with such regulations. Therefore, digital mental health services providers must work together, as they have shown they can do in the past (5, 69), to make safety a universal and non-competitive issue. The digital mental health community needs to develop cybersecurity governance that ensures that security-by-design and default is built into digital services from the onset. By creating a global cybersecurity network within digital mental health, we can collectively learn from breaches and improve our responses to future attacks with a common goal of protecting people during their most vulnerable moments. Expanding on this point, a multi-faceted approach is needed whereby regulatory organizations contribute to the development of a global cybersecurity culture within digital mental health and that mental health associations, among others, provide support for the regulations governing practitioners, service providers, and mental health facilities. Furthermore, given that digital mental health overlaps with broader aspects of digital health, including the digital therapeutics industry, it will be important to integrate and align with other frameworks, such as the Digital Therapeutic Alliance. However, it will remain critical to examine how such frameworks are integrated within mental health contexts to ensure that the most appropriate standards are being adapted and implemented in this highly sensitive space.

3. Assessing the current and future landscape

As a global community for digital mental health, we need to assess the current state of the cybersecurity landscape. For example, how have digital mental health security programs been developed to date? What are the common themes of struggle across this industry? We need to think about assessing future risks. For example, what are the risks and consequences if a cybercriminal targets medical and IoT devices that provide, transmit, and access confidential data or exploit a VR headset used for mental health and wellbeing support? Or a hacker manipulates a conversational AI system designed to offer mental health support, especially given recent occurrences of ChatGPT being hacked (70) and ChatGPT allegedly prompting a vulnerable person to a tragic outcome of death by suicide (71)? There has been a drive towards gamifying mental health treatment, but how secure is the information being gathered and stored, especially when a lot of games and apps link with virtual/social media profiles?

4. Set standards for victim support, including breach response and continuity of care procedures

While many solutions for breach responses exist from technical and operational perspectives (72), we also urgently need standards to manage disruptions in mental health care and for implementing victim support services. In the aftermath of the Vastaamo cyber-attack Finland has been actively leading the way globally on how to establish frameworks that protect victims (73). For example, the government of Finland fast-tracked legislation to allow citizens to change their personal identity codes in high-risk cases of data breaches (74). Other countries should engage in these and other forms of protecting vulnerable people.

5. Incident monitoring and establishing a central repository for reporting and archiving incidents

Cybersecurity incidents involving mental health information should be recorded, analyzed, and shared in safe ways to help the whole community respond to potential vulnerabilities. We should also be monitoring mitigation trends to deal with these threats. The attacks are coming from all types of endpoints, including some that providers can’t control or monitor.

6. More research and better budgeting: Support for cyber threat researchers, ethical hackers, and studies evaluating outcomes and impacts of data breaches

Cybercrime was predicted to cost $6 trillion in 2021 (75). By 2025, it is predicted that over $1 trillion dollars will be spent on information security (76). More digital mental health providers should be running breach and attack simulations performed by ethical hackers and they should be sharing the outcomes of these efforts. More support is needed to support and protect ethical hackers as in the past there have been occurrences whereby ethical hackers have been negatively targeted by breached organizations, for example, by threatening the individual who first notified the organization of a breach with legal action (77). We also need to better understand the immediate and long-term risks of cyber-attacks and data breaches on users and investigate the impact of interruptions to service provision on psychological outcomes. Providers must ensure there is an adequate budget for employing or consulting with cybersecurity experts, for example, having a cybersecurity officer, and/or a cybersecurity expert on their advisory board. There should also be a budget set aside for costs associated with breach responses, including to fund costs associated with offering victim support, or compensation and other aspects.

7. Identify the best methods for collecting and sharing data

Currently it is a challenge to conduct research in the field of online victimization, especially in the context of the most vulnerable, due to the lack of data. Gaining access to data sets collected by the police, such as Action Fraud, complaint records from Telecoms, or cases prosecuted under the Computer Misuse Act, could lead to more precision and evidence-based research, which could better inform policy makers.

## Data Availability

The original contributions presented in the study are included in the article/Supplementary Material, further inquiries can be directed to the corresponding author.
